# Duration of Nucleos(t)ide Analogue Treatments in Patients With Chronic Hepatitis B Virus Infection in the United States

**DOI:** 10.1111/jvh.70055

**Published:** 2025-07-16

**Authors:** Seth Anderson, Vera Gielen, Anna D. Coutinho, Laura Clark, Christopher Bell, Shayon Salehi, Renee Gennarelli, Eileen Farrelly, Dana Stafkey, Robert Gish

**Affiliations:** ^1^ GSK Collegeville Pennsylvania USA; ^2^ GSK London UK; ^3^ GSK Durham North Carolina USA; ^4^ Cencora Conshohocken Pennsylvania USA; ^5^ Hepatitis B Foundation Doylestown Pennsylvania USA

## Abstract

Viral hepatitis caused by hepatitis B virus accounts for a significant disease burden. Nucleos(t)ide analogues (NAs) are the standard of care for chronic hepatitis B (CHB) infection; however, treatment is long‐term, and viral eradication resulting in cure is rare. Adherence to NAs is vital for disease control. Here, we describe real‐world treatment patterns among adult patients with CHB infection initiating second‐generation NAs in the United States. This retrospective cohort study used United States administrative claims data. From the January 1, 2006 to July 31, 2023 period, we identified patients aged ≥ 18 years diagnosed with CHB infection who initiated second‐generation NAs. Patient characteristics and real‐world NA utilisation measures were reported, including time to discontinuation, resumption of NA treatment, adherence and predictors of adherence. In total, 6696 patients met the study eligibility criteria. Mean age was 47.2 (standard deviation: 11.5) years, and 41.6% of patients were female. The most common index NA treatments were tenofovir alafenamide (48.5%) and entecavir (41.7%). Median follow‐up duration was 24.4 months. Overall, 40.6% of patients discontinued treatment; discontinuation probability was 29.4% at 12 months and 55.6% at 5 years. Of those who discontinued, 45.7% restarted during the study period. Mean adherence (proportion of days covered [PDC]) was 0.91, and 86.5% of participants had a PDC ≥ 80%. This study highlights the challenge of long‐term persistence with NA treatment. An unmet need in CHB infection management is novel treatments with finite durations that offer an opportunity to achieve cure and mitigate disease progression.

## 1 Introduction

Viral hepatitis caused by hepatitis B virus (HBV) accounts for a significant disease burden. In 2022, an estimated 257.5 million people were living with chronic HBV (CHB) infection worldwide [1]. In the United States (US), the total prevalence of CHB infection was estimated to be as many as 2.4 million in 2018 [2]. CHB infection is defined by the seroprevalence of hepatitis B surface antigen (HBsAg) for at least six months [3]. It encompasses a spectrum of clinical conditions and can evolve to liver cirrhosis and hepatocellular carcinoma (HCC) [3, 4]. People living with CHB infection report quality‐of‐life impacts including physical (e.g., fatigue, pain, or lack of energy to work), social (e.g., stigma relating to the disease) and psychological (e.g., worry about long‐term disease progression) effects [5].

The primary aims of CHB infection treatment are to increase survival rates by mitigating the risk of liver disease progression (including both compensated and decompensated cirrhosis and HCC), lower the risk of extrahepatic cancers and inflammatory disease and enhance quality of life [6, 7, 8]. Guidelines for management of CHB infection emphasise the importance of regular screening and monitoring of liver enzymes and liver function, and viral load, as well as recommending the use of nucleos(t)ide analogues (NAs) for specific patient groups [3]. While viral replication can be effectively controlled with NAs, functional cure is rare [9]. Additionally, chronic exposure to HBV antigens such as HBsAg contributes to T‐cell exhaustion and failure to clear infection [10].

As of 2024, several NAs are approved and in use. Tenofovir disoproxil fumarate, tenofovir alafenamide and entecavir are effective first‐line treatments with low resistance rates and are preferred by the American Association for the Study of Liver Diseases (AASLD) 2018 Hepatitis B Guidance [3]. Older agents, including lamivudine, telbivudine and adefovir dipivoxil, are very rarely used due to higher rates of viral resistance and lower potency [3, 9, 11]. Surrogate disease markers such as HBV DNA and quantitative HBsAg can be used to evaluate disease stage and treatment efficacy, if indicated, and their modification or normalisation is associated with a reduced risk of cirrhosis and HCC [12]. NAs are well‐tolerated oral treatments that universally suppress HBV DNA with prolonged treatment, but their effect on HBsAg is minimal, potentially subjecting patients to continued risk of CHB‐related disease progression [9, 13].

Persistence with long‐term NA treatment is vital to achieve sustained virologic suppression, and poor adherence to NA treatment has been shown to be associated with unfavourable outcomes including viral breakthrough [14]. Poor adherence is also associated with higher mortality and greater risk of HCC and cirrhotic complications [15]. Suboptimal adherence rates are influenced by the long‐term nature of treatment, side effects and costs [9, 14, 16]. Cessation of NA treatment is associated with high rates of virologic relapse, flares of liver diseases/inflammation and risk of disease progression (e.g., hepatic decompensation) [9, 17]. It is important to understand NA treatment patterns over time to contextualise treatment discontinuation and adherence; currently, there are limited data. This study aimed to describe the duration of, and adherence to, treatment with second‐generation NAs (entecavir, telbivudine, tenofovir disoproxil fumarate and tenofovir alafenamide) among adult patients in the US with CHB infection. Predictors of treatment discontinuation and adherence were also assessed.

## 2 Methods

### 2.1 Study Design and Data Source

**FIGURE 1:**
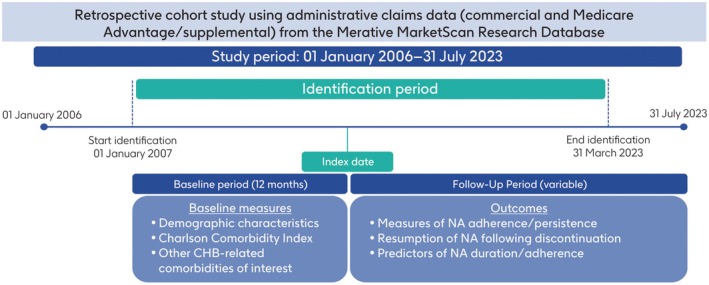
Study design. CCI, Charlson Comorbidity Index; CHB, chronic hepatitis B; NA, nucleos(t)ide analogue.

This was a retrospective cohort study using administrative claims data (commercial and Medicare Advantage/supplemental) from the Merative MarketScan Research Database (MMRD; Figure 1). The MMRD contains medical and drug claims for over 203 million individuals annually and provides information including detailed costs, utilisation, health outcomes (inpatient and outpatient services), prescription data and standard demographics [18]. The study period spanned January 1, 2006 to July 31, 2023, and the identification period was defined as January 1, 2007 to March 31, 2023. Patients were identified based on a review of their medical and pharmacy claims data. The index date was defined as the date of the first claim for a single‐drug, second‐generation NA on or after January 1, 2007, and the baseline period was defined as the 12 months (364 days not including index date) prior to the index date. The follow‐up period had to be a minimum of 4 months (120 days) and was defined from a patient's index date to the earliest of either date of onset of coinfection (human immunodeficiency virus [HIV], hepatitis D virus or hepatitis C virus), date of the end of continuous medical and pharmacy enrollment, date of skilled nursing facility or hospice admission, or data cutoff date.

### 2.2 Study Population

Patients were eligible for inclusion if they met the following criteria: had ≥ 1 pharmacy claim for a single‐drug, second‐generation NA (entecavir, telbivudine, tenofovir disoproxil fumarate or tenofovir alafenamide) during the identification period (where the date of the claim was assigned as the index date); were diagnosed with CHB infection in the 12‐month period prior to the index date (defined as at least one claim with a diagnosis code for chronic HBV infection); were aged ≥ 18 years on the index date; and were continuously enrolled in medical/pharmacy benefits for ≥ 12 months prior to, and ≥ 4 months' after, the index date. Insurance plans/payor types included point of service (POS) and exclusive provider organisation (EPO), which require referral from a primary care provider for specialist consultation, and preferred provider organisation (PPO), consumer‐driven health plan (CDHP) and high‐deductible health plan (HDHP), which do not [19]. Individuals meeting the following criteria were excluded from the analytic cohort: a claim in the baseline period with an International Classification of Diseases 10th Revision (ICD‐10) diagnosis code for HIV, hepatitis D virus, or hepatitis C virus; any NA treatment during the baseline period; and presence of > 1 unique NA on index date.

### 2.3 Study Outcomes and Data Analysis

The objectives of the study were to: (i) describe baseline demographic and clinical characteristics of patients treated with second‐generation NAs (including age, gender, US geographic region, insurance plan/payor type, Charlson Comorbidity Index [CCI]); (ii) describe real‐world time to discontinuation (TTD) of NA treatment; and (iii) describe adherence to NA treatment. Proportions of patients who restarted NA treatment after discontinuation and predictors of discontinuation and adherence were also assessed. The index date for restarting NA treatment was the date of discontinuation plus 90 days, to accurately capture each patient's at‐risk time to restart treatment following discontinuation.

TTD was defined as the time from index date to last day of treatment before discontinuation (defined as a continuous gap of ≥ 90 days without drug supply). Medication switch from one second‐generation NA to another was not considered discontinuation. Patients were censored at the end of follow‐up, including treatment discontinuation. Median TTD and the probability of discontinuing treatment at timepoints of interest were calculated using Kaplan–Meier estimation. Sensitivity analyses were performed using a treatment gap cutoff of ≥ 60 days, based on expert opinion and previous studies of treatment patterns. Adherence to treatment was assessed using proportion of days covered (PDC) by NA treatment from index date to end of follow‐up, including the date of treatment discontinuation as an additional event ending follow‐up. The intent was to understand how adherent patients were on treatment before they discontinued. PDC was calculated by dividing the total days covered by the total days of follow‐up. Switching of second‐generation NAs was allowed and counted towards the total days covered. Adherence was reported as being a PDC of ≥ 80%. Predictors of treatment discontinuation were identified using a Cox proportional hazards model with TTD as the event of interest. Predictors of adherence were identified using a logistic regression model with PDC ≥ 80% as the event of interest. Claims data were analysed to assess possible reasons for treatment discontinuation, based on frequency of clinical monitoring; these were reported as a categorical measure, being either physician guided (if the patient was under frequent or active monitoring) or non‐physician guided (if the patient was under typical or limited monitoring).

## 3 Results

**FIGURE 2:**
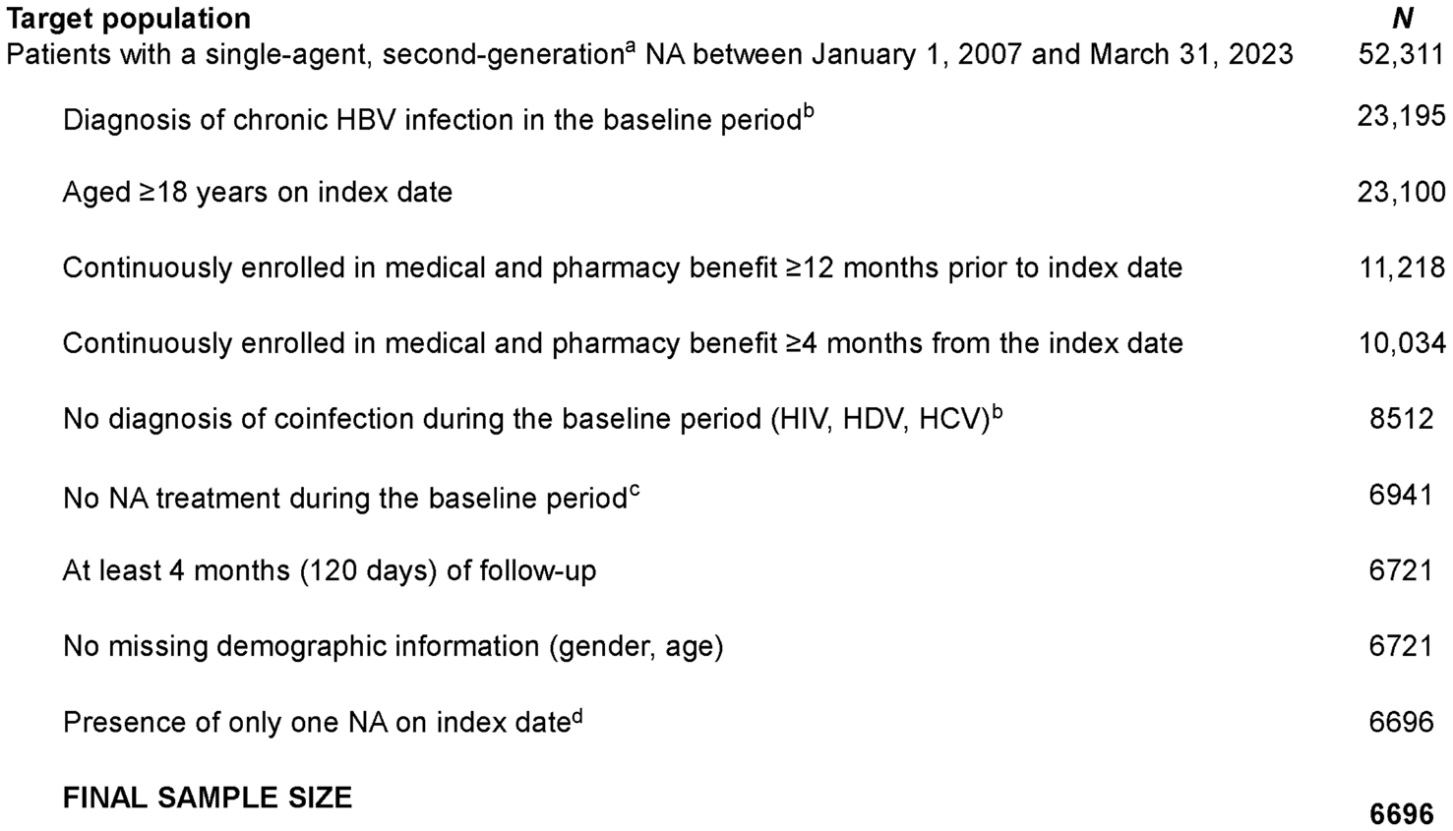
Patient flowchart. ^a^Includes entecavir, telbivudine, tenofovir disoproxil fumarate and tenofovir alafenamide. ^b^Any diagnosis in any position in the baseline period, including index date. ^c^Includes single, or a combination of, first‐ or second‐generation NAs. ^d^A total of 16 patients had more than one index NA drug; an additional 9 patients had an index NA with either adefovir or lamivudine. HBV, hepatitis B virus; HCV, hepatitis C virus; HDV, hepatitis D virus; HIV, human immunodeficiency virus; NA, nucleos(t)ide analogue.

A total of 52,311 patients receiving a single‐agent, second‐generation NA between January 1, 2007 and March 31, 2023 were identified from the MMRD. After applying the study eligibility criteria, the study population comprised 6696 patients (Figure 2).

### 3.1 Baseline Characteristics

The mean age of the final study population was 47.2 (standard deviation: 11.5) years, and 41.6% (*n* = 2787) were female (Table 1). Clinically, 76.2% (*n =* 5102) of patients had a CCI score (modified to exclude mild liver disease) of 0, and 15.7% (*n =* 1049) had a CCI score of ≥ 2. Non‐alcoholic fatty liver disease was present in 10.6% (*n =* 710) and cirrhosis in 7.4% (*n =* 493) of patients. The most common index NA treatments were tenofovir alafenamide (48.5%; *n =* 3247) and entecavir (41.7%; *n =* 2792). Median duration of follow‐up was 24.4 (interquartile range: 11.6–47.9) months.

**TABLE 1:** Baseline characteristics.

Characteristic	Overall *N* = 6696	US geographic region			
		Northeast *n* = 1309	North Central *n* = 902	South *n* = 2129	West *n* = 2323
Age (years), mean (SD)	47.2 (11.5)	47 (12.0)	47.9 (12.8)	47.1 (11.5)	47.1 (10.7)
Age group (years), *n* (%)					
18–34	946 (14.1)	203 (15.5)	147 (16.3)	296 (13.9)	297 (12.8)
35–49	2885 (43.1)	571 (43.6)	351 (38.9)	900 (42.3)	1047 (45.1)
50–64	2519 (37.6)	439 (33.5)	327 (36.3)	841 (39.5)	899 (38.7)
65+	346 (5.2)	96 (7.3)	77 (8.5)	92 (4.3)	80 (3.4)
Female, *n* (%)	2787 (41.6)	554 (42.3)	341 (37.8)	869 (40.8)	1011 (43.5)
Plan type, *n* (%)					
FFS	161 (2.4)	15 (1.1)	65 (7.2)	60 (2.8)	18 (0.8)
EPO/PPO	3435 (51.3)	767 (58.6)	479 (53.1)	1197 (56.2)	972 (41.8)
HMO	1565 (23.4)	181 (13.8)	132 (14.6)	323 (15.2)	923 (39.7)
POS	600 (9.0)	185 (14.1)	66 (7.3)	224 (10.5)	122 (5.3)
CDHP/HDHP	818 (12.2)	124 (9.5)	143 (15.9)	290 (13.6)	260 (11.2)
Unknown	117 (1.7)	37 (2.8)	17 (1.9)	35 (1.6)	28 (1.2)
Index year, *n* (%)					
2007	185 (2.8)	23 (1.8)	27 (3.0)	65 (3.1)	69 (3.0)
2008	303 (4.5)	35 (2.7)	43 (4.8)	124 (5.8)	101 (4.3)
2009	498 (7.4)	75 (5.7)	87 (9.6)	174 (8.2)	159 (6.8)
2010	541 (8.1)	96 (7.3)	81 (9.0)	163 (7.7)	200 (8.6)
2011	539 (8.0)	113 (8.6)	53 (5.9)	154 (7.2)	216 (9.3)
2012	654 (9.8)	132 (10.1)	71 (7.9)	193 (9.1)	252 (10.8)
2013	545 (8.1)	106 (8.1)	67 (7.4)	144 (6.8)	224 (9.6)
2014	486 (7.3)	119 (9.1)	57 (6.3)	132 (6.2)	173 (7.4)
2015	418 (6.2)	95 (7.3)	46 (5.1)	131 (6.2)	145 (6.2)
2016	477 (7.1)	102 (7.8)	64 (7.1)	151 (7.1)	158 (6.8)
2017	470 (7.0)	98 (7.5)	68 (7.5)	166 (7.8)	136 (5.9)
2018	386 (5.8)	88 (6.7)	48 (5.3)	135 (6.3)	114 (4.9)
2019	319 (4.8)	56 (4.3)	57 (6.3)	98 (4.6)	107 (4.6)
2020	272 (4.1)	61 (4.7)	41 (4.5)	99 (4.7)	69 (3.0)
2021	253 (3.8)	49 (3.7)	35 (3.9)	79 (3.7)	89 (3.8)
2022	286 (4.3)	54 (4.1)	44 (4.9)	93 (4.4)	95 (4.1)
2023^a^	64 (1.0)	7 (0.5)	13 (1.4)	28 (1.3)	16 (0.7)
CCI score^b^, *n* (%)					
0	5102 (76.2)	984 (75.2)	636 (70.5)	1603 (75.3)	1855 (79.9)
1	545 (8.1)	101 (7.7)	73 (8.1)	191 (9.0)	176 (7.6)
2+	1049 (15.7)	224 (17.1)	193 (21.4)	335 (15.7)	292 (12.6)
CCI score, mean (SD)	0.6 (1.5)	0.7 (1.6)	0.9 (1.7)	0.7 (1.5)	0.5 (1.3)
Cirrhosis during baseline, *n* (%)	493 (7.4)	75 (5.7)	96 (10.6)	195 (9.2)	123 (5.3)
HCC during baseline, *n* (%)	96 (1.4)	18 (1.4)	16 (1.8)	29 (1.4)	32 (1.4)
Liver failure during baseline, *n* (%)	137 (2.0)	26 (2.0)	24 (2.7)	53 (2.5)	33 (1.4)
Liver transplantation during baseline, *n* (%)	46 (0.7)	8 (0.6)	7 (0.8)	19 (0.9)	10 (0.4)
NAFLD during baseline, *n* (%)	710 (10.6)	138 (10.5)	81 (9.0)	262 (12.3)	227 (9.8)
Index NA treatment, *n* (%)					
Entecavir	2792 (41.7)	475 (36.3)	348 (38.6)	923 (43.4)	1037 (44.6)
Telbivudine	41 (0.6)	12 (0.9)	6 (0.7)	14 (0.7)	9 (0.4)
Tenofovir disoproxil fumarate	616 (9.2)	117 (8.9)	96 (10.6)	242 (11.4)	160 (6.9)
Tenofovir alafenamide	3247 (48.5)	705 (53.9)	452 (50.1)	950 (44.6)	1117 (48.1)
Median follow‐up^c^ (months)	24.4	22.7	25.4	21.9	27.9
(Q1, Q3)	(11.6, 47.9)	(10.9, 45.1)	(11.3, 49.5)	(10.6, 43.0)	(13.2, 53.5)
Minimum, maximum	4, 193.2	4, 182	4, 193.2	4, 186.3	4, 189.1

Abbreviations: CCI, Charlson Comorbidity Index; CDHP, consumer‐driven health plan; EPO, exclusive provider organisation; FFS, fee‐for‐service; HCC, hepatocellular carcinoma; HDHP, high deductible health plan; HMO, health maintenance organisation; NA, nucleos(t)ide analogue; NAFLD, non‐alcoholic fatty liver disease; POS, point of service; PPO, preferred provider organisation; Q, quartile; SD, standard deviation.

^a^ The identification period ended on March 31, 2023.

^b^ CCI score does not include mild liver disease, since all patients had hepatitis B, which is part of this comorbidity.

^c^ End of follow‐up defined as the earlier of the date of onset of coinfections, date of the end of continuous medical and pharmacy enrolment, date of skilled nursing facility or hospice admission, or data cutoff date.

### 3.2 Treatment Discontinuation

Overall, 40.6% of study participants discontinued their second‐generation NA treatment (Table 2). The probability of treatment discontinuation within 12 months of starting NA treatment was 29.4%, increasing to 55.6% within 5 years (Table 2). The median TTD was 45 months (Table 2). The proportion of participants discontinuing treatment, the median TTD and the probability of discontinuing treatment all showed variation by US geographic region (Table 2). In the sensitivity analysis, 46.9% of study participants discontinued their treatment. Among those who discontinued, only 12.6% did so due to a possible physician‐guided decision (based on evidence that the patient was under frequent or active monitoring defined as ≥ 3 claims for a comprehensive metabolic panel and/or ≥ 3 claims for an HBV DNA test within 90 days before or after discontinuation).

**TABLE 2:** Patterns of treatment discontinuation (overall and by US geographic region).

Characteristic	Overall *N* = 6696	US geographic region			
		Northeast *n* = 1309	North Central *n* = 902	South *n* = 2129	West *n* = 2323
TTD (months) using Kaplan–Meier methods, median (95% CI)	45 (41–49)	54 (46–61)	47 (35–59)	31 (27–37)	54 (48–64)
Probability of discontinuing treatment, % (95% CI)					
12 months	29.4 (28.3–30.6)	29.9 (27.4–32.6)	30.0 (27.0–33.3)	32.6 (30.5–34.7)	26.3 (24.5–28.2)
24 months	39.5 (38.2–40.8)	39.6 (36.6–42.7)	40.0 (36.5–43.8)	45.4 (42.9–47.9)	34.4 (32.3–36.6)
36 months	45.8 (44.3–47.3)	43.4 (40.2–46.8)	46.8 (42.9–50.9)	52.3 (49.6–55.1)	41.2 (38.8–43.6)
60 months	55.6 (53.8–57.4)	52.7 (48.6–57.0)	55.1 (50.4–60.0)	62.7 (59.3–66.1)	51.3 (48.5–54.3)

Abbreviations: CI, confidence interval; TTD, time to discontinuation; US, United States.

### 3.3 Resumption of Treatment

Among patients who discontinued index NA treatment, 45.7% restarted a second‐generation NA at some point during the study period (Table 3). The probability of resuming treatment was 43.7% by 12 months post‐discontinuation, increasing to 56.5% by 3 years (Table 3). The number of patients who did not resume any NA treatment after discontinuation (*n =* 1478) corresponds to 22% of the overall study cohort.

**TABLE 3:** Duration and resumption of NA treatment among patients who discontinued treatment.

Characteristic	Overall *N* = 6696
Discontinued second‐generation NA treatment, *n* (%)	2721 (40.6)
Duration of second‐generation NA treatment (months), mean (SD)	12.8 (17.8)
Median (Q1, Q3)	5.9 (2.0, 15.3)
Crude treatment resumption proportion among those who discontinued, *n* (%)	1243 (45.7)
Probability of restarting treatment^a^, % (95% CI)	
6 months	34.0 (32.2–35.9)
12 months	43.7 (41.7–45.8)
24 months	51.2 (49.0–53.4)
36 months	56.5 (54.1–59.0)

Abbreviations: CI, confidence interval; NA, nucleos(t)ide analogue; Q, quartile; SD, standard deviation; TTD, time to discontinuation.

^a^ TTD to restarting treatment censored at end of follow‐up. The index date was the date of discontinuation plus 90 days, to accurately capture each patient's at‐risk time to restart treatment following discontinuation.

### 3.4 Adherence

From index date to end of follow‐up, mean PDC was 0.91, and 86.5% of patients had a PDC ≥ 80% (Table 4). Among those with follow‐up of 12 months, 2 years and 3 years, mean PDC was 0.92, 0.92 and 0.93, respectively. PDC ≥ 80% was 90.4% at 12 months, 91.5% at 2 years and 94.6% at 3 years among patients who had follow‐up through the timepoint of interest (Table 4).

**TABLE 4:** Adherence to NA treatment^a^ (overall).

Characteristic	Overall *N =* 6696	
	*n* (%)	Mean (SD)
PDC from index date to:		
End of follow‐up	—	0.91 (0.12)
6 months^b^	4872 (72.8)	0.92 (0.12)
12 months^b^	3444 (51.4)	0.92 (0.09)
24 months^b^	2028 (30.3)	0.92 (0.08)
36 months^b^	1307 (19.5)	0.93 (0.07)
Rate of adherent patients (PDC ≥ 80%) from index date to:		
End of follow‐up	5791 (86.5)	—
6 months^b^	4253 (87.3)	—
12 months^b^	3112 (90.4)	—
24 months^b^	1855 (91.5)	—
36 months^b^	1236 (94.6)	—

Abbreviations: NA, nucleos(t)ide analogue; PDC, proportion of days covered; SD, standard deviation.

^a^ For these measures of adherence, the end of follow‐up was defined as the earlier of the date of treatment discontinuation, date of onset of coinfections, date of the end of continuous medical and pharmacy enrolment, date of skilled nursing facility or hospice admission, or data cutoff date.

^b^ Evaluated only among patients who have follow‐up through the timepoint of interest.

### 3.5 Predictors of Discontinuation and Adherence

Those who were significantly more likely (*p* < 0.05) to be adherent to treatment were older patients, males, those enrolled in POS plan versus an EPO or PPO, those with cirrhosis (compensated and decompensated), those with shorter follow‐up in months and those receiving tenofovir alafenamide as index treatment versus entecavir. Alternatively, younger patients, females, those enrolled in a CDHP or HDHP plan versus those enrolled in an EPO/PPO, those from the South versus the West, those with higher CCI scores and those with longer follow‐up in months were significantly less likely to be adherent (Table 5).

**TABLE 5:** Significant predictors (*p* < 0.05) of adherence and discontinuation.

Adherence (PDC ≥ 80%)		Treatment discontinuation	
Characteristic	OR (95% CI)^a^	Characteristic	HR (95% CI)^b^
Age at start of treatment	1.01 (1.00–1.01)	Age at start of treatment	0.99 (0.99–1.00)
Gender [female vs. male]	0.82 (0.75–0.91)	Gender [female vs. male]	1.15 (1.07–1.24)
CCI score^c^	0.91 (0.87–0.94)	CCI score^c^	1.07 (1.04–1.10)
Index NA treatment [tenofovir alafenamide vs. entecavir]	1.83 (1.48–2.25)	Index NA treatment [tenofovir alafenamide vs. entecavir]	0.62 (0.52–0.73)
US geographic region [South vs. West]	0.62 (0.55–0.70)	Index NA treatment [telbivudine vs. entecavir]	1.68 (1.10–2.56)
Cirrhosis	1.38 (1.13–1.69)	Geographic region [South vs. West]	1.35 (1.23–1.48)
Duration of follow‐up	0.99 (0.99–1.00)	Cirrhosis	0.79 (0.67–0.93)
Plan type [POS vs. EPO/PPO]	1.28 (1.07–1.53)	HCC	0.68 (0.47–0.99)
Plan type [CDHP/HDHP vs. EPO/PPO]	0.80 (0.68–0.94)	Plan type [POS vs. EPO/PPO]	0.87 (0.75–1.00)
Index year [2020 vs. 2007]	0.84 (0.57–1.25)	Plan type [CDHP/HDHP vs. EPO/PPO]	1.15 (1.02–1.30)
Index year [2023 vs. 2007]	2.00 (1.04–3.83)	Index year [2015 vs. 2007]	0.76 (0.59–0.99)

Abbreviations: CCI, Charlson Comorbidity Index; CDHP, consumer‐driven health plan; CI, confidence interval; EPO, exclusive provider organisation; HCC, hepatocellular carcinoma; HDHP, high deductible health plan; HR, hazard ratio; NA, nucleos(t)ide analogue; OR, odds ratio; PDC, proportion of days covered; POS, point of service; PPO, preferred provider organisation; TTD, time to discontinuation; US, United States.

^a^ ORs represent odds of adherence (PDC ≥ 80%). Values > 1 indicate a factor is associated with increased adherence relative to the referent group, and values < 1 indicate a factor is associated with decreased adherence relative to the referent group.

^b^ HRs represent risk of treatment discontinuation. Values > 1 indicate a factor is associated with shorter TTD (i.e., higher risk of discontinuation) relative to the referent group, and values < 1 indicate a factor is associated with longer TTD (i.e., lower risk of discontinuation) relative to the referent group.

^c^ CCI score does not include mild liver disease, since all patients had hepatitis B, which is part of this comorbidity.

Significant (*p* < 0.05) predictors of discontinuation included age, gender, plan type, US geographic region, CCI score, cirrhosis, HCC and index NA treatment. Older patients, males, those enrolled in a POS plan, those with cirrhosis, those with HCC and those receiving tenofovir alafenamide as index treatment were significantly less likely to discontinue treatment. Alternatively, younger patients, females, those enrolled in a CDHP or HDHP, those from the South, those with higher CCI scores and those receiving telbivudine as index treatment were significantly more likely to discontinue treatment (Table 5).

## 4 Discussion

CHB infection is generally underdiagnosed, as the condition is typically asymptomatic [20]. Nevertheless, there is a substantial disease burden associated with disease progression, including cirrhosis, HCC and death [7]. Treatment of CHB infection is made difficult by persistent viral replication and immunosuppressive effects, and functional cure is rare [9, 10]. While NAs can control viral replication, treatment is long‐term; adherence to and persistence with treatment regimens is therefore critical [3].

This study assessed treatment discontinuation and adherence with second‐generation NAs among adult patients in the US with CHB infection. Baseline demographic and clinical characteristics of the final study sample were consistent with previously published data [21]. We also found 7.4% of patients to have a history of cirrhosis, consistent with other findings [21]. Tenofovir alafenamide was the most frequently used NA treatment, followed by entecavir.

Given that successful NA treatment in patients with CHB infection requires long‐term treatment to ensure viral DNA suppression, it is important to note the relatively high rate of treatment discontinuation (~40%) observed in this study, and that ~20% of patients did not restart treatment; this may indicate a need for additional support to help patients continue with their NA treatment. A substantial proportion of patients (45.7%) in our study who discontinued eventually resumed treatment. This may suggest that virologic or biochemical relapse often occurs after treatment discontinuation, and highlights an unmet need for treatments of shorter and finite duration that can achieve functional cure. Previous studies have suggested that as many as 94% of patients may experience virologic relapse following NA treatment cessation [17]. In a systematic literature review, high‐risk events including liver failure, HCC and death were highlighted as potential consequences of virologic relapse following cessation of NA treatment, as well as risk of drug resistance following treatment resumption [22].

In our study of patients on second‐generation NA treatment, adherence as indicated by PDC data was relatively high. At the end of follow‐up (inclusive of treatment discontinuation), patients had (on average) 91% of days covered by a prescription. These data on adherence are consistent with those from a similar claims‐based study that also used PDC as a measure of adherence [23]. Average adherence among patients was 87.8% of days covered, with higher adherence observed in existing patients compared with newly diagnosed patients [23]. Other studies have highlighted patient preference (e.g., dose or administration route) being an important factor affecting adherence [24]. Adherence to NAs appears to be higher compared with treatments for other chronic diseases. In a retrospective study using PDC to assess adherence to antiretroviral medication in adults in the US with HIV, approximately 60% of patients had suboptimal adherence (PDC < 90%), with an average PDC of 74% over a 12‐month follow‐up period [25]. Of patients with type 2 diabetes newly initiating oral semaglutide or a dipeptidyl peptidase‐4 inhibitor, around 42% of patients had a PDC ≥ 80% over a 12‐month follow‐up period [26].

There was a high degree of similarity among predictors of treatment discontinuation and adherence (e.g., older age at treatment start, female gender, lower CCI score, use of tenofovir alafenamide versus entecavir, US geographic region [South versus West], cirrhosis), reflecting the intertwined relationship between the two. Similar to our study, older age at time of treatment has previously been shown to predict better adherence to NA treatment [23]. It is possible that female gender was a predictor of discontinuation due to pregnancy, since 14% of patients were 18–34 (25% were ≤ 39 years of age) and 41% of the cohort was female. However, we do not anticipate this being a main driver considering antivirals, such as TDF, are generally considered safe in pregnancy throughout the literature [27]. The impact of plan type may reflect underlying social determinants of health. CDHP/HDHP plans tend to shift more upfront cost burden to patients through higher deductibles and out‐of‐pocket expenses, which may lead to cost‐related non‐adherence or discontinuation, whereas patients on POS plans are more likely to have care coordination and primary care engagement due to the need for referral.

The impact of high CCI on discontinuation may suggest that patients with competing health priorities face more immediate health concerns and thus deprioritise CHB infection management as compared to higher priority conditions such as heart failure or cancer. It may also suggest that patients with higher CCI are seen by multiple specialists, receive many medications and have more complex care coordination which could lead to treatment discontinuation due to drug interaction concerns and the implications of fragmented care where there is a lack of a clear ‘owner’ of CHB infection management. Lastly, patients with higher CCI may also be more likely to have socioeconomic disadvantages or limited access to specialty care that would affect disease management. Among those who discontinued treatment in our study, only 13% had evidence that they were under frequent or active monitoring. This suggests that the majority of discontinuations were not guided by physicians or based on serologic findings. Cessation of NA therapy is associated with high rates of relapse, which can happen soon or many years after stopping treatment [17]. The latest guidelines and clinical evidence emphasise the importance of adherence to CHB infection medication such as NAs to achieve the best clinical outcomes, and monitoring is important to minimise risk of relapse in patients who do discontinue treatment [3, 17]. This study contributes to our understanding of NA treatment in CHB infection treatment, and lays a foundation for refining care strategies aimed at improving long‐term outcomes in the complex landscape of CHB infection management.

### 4.1 Limitations

Potential limitations of our study should be considered. Since the study was descriptive in nature, causal inferences could not be drawn. Also, time‐to‐event analyses such as Kaplan–Meier assume censoring is non‐informative; however, informative censoring may be present in the study due to censoring endpoints being directly related to outcomes of interest, such as entry to hospice or presence of coinfection. However, in the case of coinfection, it is likely that patients would no longer be treated with a single‐drug NA. Additionally, all TTD analyses are based on the definition of discontinuation being a continuous gap of 90 days without NA drug supply. It is possible that treatment discontinuation was misclassified based on the chosen discontinuation gap, but to mitigate possible bias arising from this, a sensitivity analysis using a gap period of 60 days was also conducted.

Our study utilised claims data, with patients identified and classified based on the presence of billing codes, meaning there is a possibility of measurement error or misclassification of disease and treatment exposure. This is particularly relevant given the study period and the switch from the ICD‐9 coding system to ICD‐10. Pharmacy claims also provide information on filled prescriptions, but not necessarily on the actual use of medication. NA adherence could therefore have been overestimated. Possession‐based refill measures such as the PDC do not fully capture dynamic changes in adherence patterns over time, and may not account for variations in prescription duration, changes in dosing frequency or irregular patient behaviour. Additionally, given the observational nature of this study, it is difficult to ascertain specific reasons for discontinuation, specifically in the absence of clinical notes. Finally, results may only be generalisable to insured patients in the US who are aged ≥ 18 years with CHB infection and receiving second‐generation NA treatment without the presence of coinfections.

## 5 Conclusions

Treatment discontinuation is a key challenge for long‐term NA treatment in patients with CHB infection. This study also suggests that there is an unmet need for additional support to help patients persist with NA treatment. In addition, study findings highlight the need for novel CHB infection treatments with shorter and finite treatment durations that offer an opportunity to achieve cure with the elimination of HBsAg. Such treatments may help mitigate the high discontinuation rate and need for rigid monitoring that are associated with currently available treatments.

## Author Contributions

All authors made a significant contribution to the work reported, whether that was in the conception, study design, execution, acquisition of data, analysis and interpretation or in all these areas; took part in drafting, revising or critically reviewing the manuscript; gave final approval of the version to be published; have agreed on the journal to which the article has been submitted; and agree to be accountable for all aspects of the work.

## Disclosure

Authorship: All named authors meet the International Committee of Medical Journal Editors (ICMJE) criteria for authorship for this article, take responsibility for the integrity of the work as a whole and have given their approval for this version to be published.

## Ethics Statement

This study complied with all applicable laws regarding subject privacy. No direct subject contact or primary collection of individual human subject data occurred. Study results are in tabular form and presented as aggregate analyses that omit subject identification; therefore, informed consent and ethics committee or institutional review board approval was not required. Subject identifiers are not included.

## Conflicts of Interest

S.A., V.G. and L.C. were employed by and held financial equities in GSK (at time of study). A.D.C., C.B. and S.S. are employed by and hold financial equities in GSK. R.G., E.F. and D.S. are employed by Cencora, which received research funds from GSK for this study. R.G. has received grants/research support from Gilead in the last 2 years and has performed as a consultant and/or advisor to Abacus, Abbott, AbbVie, Albireo, Aligos, Altimmune, Antios, Arrowhead, AstraZeneca, Audentes Therapeutics, Corcept, Dynavax, Effectus, Eiger, Eisai, ENYO, Genentech, Genlantis, Gerson Lehrman Group, Gilead Sciences, GSK, Helios, HepaTx, HepQuant, Intercept, Ipsen, Janssen, JBS Science, Kinnate Biopharma, Merck, Pfizer, Precision BioSciences, Seres Therapeutics, Topography Health, Tune Therapeutics, Venatorx and Virion in the last 2 years.

## Data Availability

The data that support the findings of this study are available from the corresponding author upon reasonable request.
